# Medicare Part D Use and Costs for Immune-Mediated Neurologic Therapies

**DOI:** 10.1001/jamanetworkopen.2025.38277

**Published:** 2025-10-20

**Authors:** Ka-Ho Wong, Esther Zeng, Tammy L. Smith, Jordan B. King, Erica Marini, Abigail Sorenson, Heewon Hwang, Chloe Stein, Trieste Francis, John W. Rose, Vivek K. Reddy, Adam Helme de Havenon, Stacey L. Clardy

**Affiliations:** 1 Department of Neurology, University of Utah School of Medicine, Salt Lake City; 2 Department of Population Health Science, University of Utah School of Medicine, Salt Lake City; 3 Department of Undergraduate Studies, University of California, Berkeley; 4 Veterans Affairs Health System, Salt Lake City Health Care, Salt Lake City, Utah; 5 Institute of Health Research, Kaiser Permanente Colorado, Aurora; 6 University of Utah School of Pharmacy, Salt Lake City; 7 Department of Neurology, Yale University, New Haven, Connecticut

## Abstract

**Importance:**

The expansion in the number of disease-modifying therapies (DMTs) for immune-mediated neurologic diseases raises critical questions about trends in their use and cost over time and whether this growth could be contributing to escalating financial burdens within the Medicare system.

**Objective:**

To examine trends in claims and Medicare Part D payments for DMTs for immune-mediated neurologic diseases.

**Design and Setting:**

This retrospective economic evaluation analyzed Medicare Part D Prescriber Public Use Files from January 1, 2013, to December 31, 2022, restricting to prescription drug claims categorized under the clinician-defined neurology taxonomy. Only prescription drugs used as DMTs for multiple sclerosis, neuromyelitis optica spectrum disorder, and other immune-mediated neurologic disorders were included. To ensure accuracy, these medications were validated by 2 pharmacists. The data were analyzed between October 1, 2023, and November 12, 2024.

**Main Outcomes and Measures:**

Ten-year trends in total payments, number of claims, and payment per claim for DMTs were assessed for their contribution to overall cost increases. Inflation adjustments were applied using both medical care–specific and prescription drug–specific indices.

**Results:**

Between 2013 and 2022, 63 unique drugs used as DMTs for multiple sclerosis, neuromyelitis optica spectrum disorder, and other immune-mediated neurologic disorders (eg, autoimmune encephalitis, myasthenia gravis) accounted for 6 526 278 claims and contributed $35 billion in Medicare drug expenditures. Between 2013 and 2022, annual claims for DMTs increased by 3.8% (from 549 766 to 570 298 claims). However, total payments for DMTs saw a disproportionate increase of 70.3% (95% CI, 13.5%-130.3%) from 2013 to 2022. After adjusting for medical care or prescription drug inflation, 2022 payments per claim increased by 29.1% (95% CI, 15.6% to 51.6%) and 35.9% (95% CI, 29.5%-50.3%), respectively, indicating that the rise in drug costs exceeded adjustments for medical care or prescription drug inflation rates. For the 29 DMTs available over the entire 10-year period, total claims decreased by 14.8% (from 546 026 to 465 089). Despite this reduction in claims, total payments increased by 60.5% (95% CI, 50.9%-90.6%). After adjusting for medical care inflation, the total payment increased significantly by 26.2% (95% CI, 10.5%-49.4%).

**Conclusions and Relevance:**

Over the past decade, Medicare Part D expenditures for DMTs targeting immune-mediated neurologic diseases have increased substantially, despite relatively stable claim volumes. These findings show that increases have consistently outpaced inflation rates, adding substantial economic pressure to federally funded neurologic care.

## Key Points

**Question:**

What were the trends in the use of and Medicare Part D payments for disease-modifying therapies (DMTs) for immune-mediated neurologic diseases from 2013 to 2022?

**Findings:**

In this economic analysis of 63 unique drugs used for DMTs, those for immune-mediated neurologic diseases accounted for a 3.8% increase in total Medicare Part D claims; however, total payments for these therapies rose disproportionately by 70.3%, with expenditures significantly exceeding estimated medical care and prescription drug inflation adjustments.

**Meaning:**

Although DMT use for immune-mediated neurologic diseases has remained stable, associated costs have increased substantially beyond inflation, suggesting other contributors to rising expenditures.

## Teaser

This economic evaluation examines trends in claims and Medicare Part D payments for disease-modifying therapies for multiple sclerosis, neuromyelitis optica spectrum disorder, and other immune-mediated neurologic disorders.

## Introduction

The rising cost of prescription medications for neurologic disease is a major concern, with the Centers for Medicare & Medicaid Services bearing much of the financial burden. For multiple sclerosis, disease-modifying therapy (DMT) costs have grown at a rate 5 times greater than inflation and now account for more than half of total multiple sclerosis care expenditures.^1,2,3,4,5,6^ Between 2019 and 2022, the US Food and Drug Administration (FDA) approved the first 3 DMTs for neuromyelitis optica spectrum disorder (NMOSD), with mean annual per-patient costs ranging from $122 850 to $728 136.^7,8,9,10,11^ Many of these therapies are also used beyond multiple sclerosis and NMOSD, including myasthenia gravis, autoimmune encephalitis, stiff person spectrum disorders, and other immune-mediated neurologic diseases.^12,13,14,15^

With growing recognition of these disorders and expanded availability of DMTs, it is essential to assess how use and costs have changed over time. This study evaluates a decade of inflation-adjusted Medicare Part D data through 2022 to examine trends in claims, total payments, and per-claim costs for DMTs used to treat immune-mediated neurologic diseases. By examining a broad range of neurologic DMTs beyond multiple sclerosis and NMOSD, we aimed to provide a comprehensive view of national spending patterns and the evolving economic burden these therapies place on federally funded neurologic care.

## Methods

### Data Source

This economic evaluation is a retrospective analysis of prescription drug claims using the 2013 to 2022 Medicare Part D Prescriber Public Use Files. These files contain annual Medicare beneficiary drug claims linked to clinicians with a National Provider Identifier (excluding drugs with ≤10 claims).^16^ Local institutional review board approval and informed consent were not required for the analysis of deidentified, publicly available data, in accordance with the Common Rule. This study adhered to the Consolidated Health Economic Evaluation Reporting Standards (CHEERS) reporting guideline.

To ensure that the use of DMTs is appropriately aligned with neurologic conditions, the data selection process was restricted to entries categorized under the neurology taxonomy as defined by clinicians, within the Medicare Part D Prescriber Public Use Files. These files reflect medications reimbursed through Medicare Part D, including many infusible therapies that are increasingly dispensed through specialty pharmacies. As such, the majority of therapies included in this analysis, regardless of route of administration, were covered under Medicare Part D. Prescription drug names for DMTs used in immune-mediated neurologic disorders were verified independently by 2 pharmacists (J.B.K. and E.M.) (eTable 1 in Supplement 1). Discrepancies were resolved through consensus to ensure accuracy and consistency in the dataset.

### Outcomes

The primary outcomes of this study were the total annual number of claims and payments for DMTs and the mean payment per DMT claim. The primary analysis included all DMT payments and claims from January 1, 2013, to December 31, 2022, to assess the cost burden on the Centers for Medicare & Medicaid Services and the contribution of DMT use for immune-mediated neurologic disorders (by drug type and prescriber specialty) among Medicare beneficiaries. The secondary analysis examined 10-year trends after excluding DMTs introduced after 2013 or without claims in all study years, allowing for consistent longitudinal comparisons. Percentage change in payments was calculated as follows: ([2022 Medicare payment − 2013 Medicare payment]/2013 Medicare payment) × 100, with the same equation applied to the payment per claim.^17^ We also performed a secondary analysis based on the route of administration and class of DMT. Drug class adjudications and indications were reviewed by a neuroimmunologist (J.W.R.) and 2 pharmacists (J.B.K. and E.M.), with discrepancies resolved by consensus. This approach provided a detailed assessment of the financial landscape of DMT use and its evolution over time.

### Inflation Adjustment

To assess whether the observed increase in DMT payments was attributable to factors beyond inflation, we conducted an analysis that adjusted for 2 specific inflation rates: (1) medical care and (2) prescription drug. This approach allowed for a more nuanced understanding of the underlying drivers of payment changes, distinguishing between inflationary trends and other contributing factors. To account for these inflation rates, we used the US Bureau of Labor Statistics Consumer Price Index database.^18^ The medical care inflation rate tracks changes in health care costs over time, including hospital services, physician fees, and medical supplies, while the prescription drug inflation rate measures price changes for medications purchased through retail, mail-order, or online pharmacies. Both metrics, subsets of the Consumer Price Index, provide insights into how health care and pharmaceutical costs evolve compared with other economic sectors.^19^ The inflation-adjusted payments for DMTs were calculated using 2013 as the reference year to account for changes in the value of currency over time.

### Statistical Analysis

Descriptive statistics are reported for total annual payments, number of claims, and payments per claim, stratified by actual cost and costs adjusted for medical care and prescription drug inflation (in 2013 US dollars). Trends over time were assessed using linear regression models, with year as the independent variable. Percentage changes in claims and payments from 2013 to 2022 were calculated, and *P* values for trends were derived using Wald tests.

Comparisons across DMT routes of administration and therapeutic subclasses were evaluated using analysis of variance. Differences in inflation-adjusted costs between 2013 and 2022 were assessed using paired *t* tests. All statistical tests were 2-sided with significance set at *P* < .05. Analyses were performed between October 1, 2023, and November 12, 2024, using Stata, version 17.0 (StataCorp LLC).

## Results

Between 2013 and 2022, a total of 269 478 912 claims for neurologic diseases were processed, amounting to a cumulative expenditure of $85.9 billion. Within these claims, 63 unique DMTs (37 branded and 26 generic) specific to multiple sclerosis, NMOSD, and other immune-mediated neurologic disorders accounted for 2.4% of claims and contributed $35 billion (40.8%) in Medicare drug expenditures.

### Claims and Payment for All DMTs

**Figure 1.**
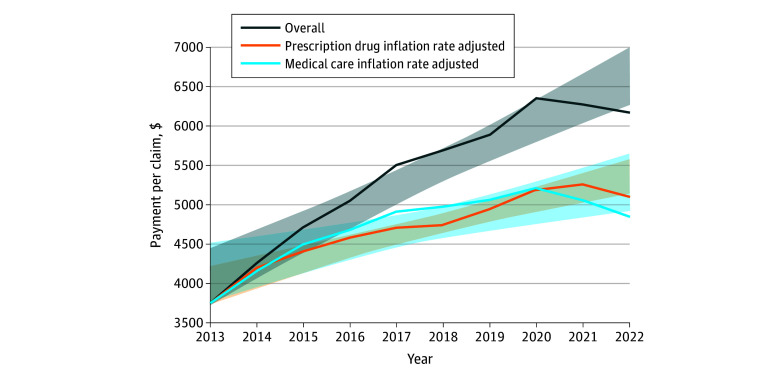
Trends in Mean Payment Per Claim for Immune-Mediated Neurologic Disease-Modifying Therapies (63 Unique), 2013 to 2022 Shading indicates the 95% CI.

The number of claims increased 3.8% (from 549 766 in 2013 to 570 298 in 2022). In contrast, total costs significantly increased 70.3% (95% CI, 13.5%-130.3%; *P* = .02). Payment per claim increased significantly by 64.1% (95% CI, 57.2%-93.6%; *P* < .001). After adjusting for inflation, the 2022 payment per claim increased by 29.1% (95% CI, 15.6%-51.6%; *P* = .003) when accounting for medical care inflation and by 35.9% (95% CI, 29.5%-50.3%; *P* < .001) when accounting for prescription drug inflation (Table 1; Figure 1).

**Table 1.** All Claims and Payments for All and Continuously Available DMTs, 2013 to 2022

Variable	For all DMTs (63 unique)				For continuously available DMTs (29 unique)				Total Medicare payments for all DMTs (63 unique), million $		Percentage change (95% CI)	*P* value
	Mean (SD) payment per eligible claim, $		Percentage change (95% CI)	*P* value	Mean (SD) payment per eligible claim, $		Percentage change (95% CI)	*P* value				
	2013 (n = 549 766)	2022 (n = 570 298)			2013 (n = 546 026)	2022 (n = 465 089)			2013 (n = 549 766)	2022 (n = 570 298)		
Overall	3754.78 (2390.92)	6163.34 (6029.50)	64.2 (57.2 to 93.6)	<.001	3750.82 (2398.13)	6020.03 (5031.92)	60.5 (50.9 to 90.6)	<.001	2064.25	3514.94	70.3 (13.5 to 130.3)	.02
Medical care inflation rate adjusted	3754.78 (2390.92)	4845.99 (4740.75)	29.1 (15.6 to 51.6)	.003	3750.82 (2398.13)	4733.31 (3956.40)	26.2 (10.5 to 49.4)	.007	2064.25	2763.66	33.9 (−28.3 to 82.12)	.29
Prescription drug inflation rate adjusted	3754.78 (2390.92)	5103.79 (4992.95)	35.9 (29.5 to 50.3)	<.001	3750.82 (2398.13)	4985.11 (4166.87)	32.9 (24.8 to 47.3)	<.001	2064.25	2910.68	41.0 (−11.95 to 79.53)	.13

Abbreviation: DMT, disease-modifying therapy.

### Claims and Payment for Continuously Available DMTs

**Figure 2.**
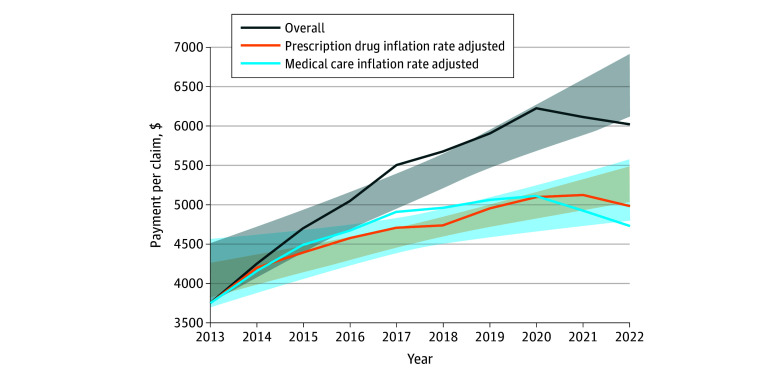
Trends in Mean Payment Per Claim for 29 Unique Immune-Mediated Neurologic Disease-Modifying Therapies That Were Available Consistently, 2013 to 2022 Shading indicates the 95% CI.

From 2013 to 2022, 29 consistently documented DMTs (16 branded and 13 generic) generated 6.2 million claims and $32.7 billion in Medicare expenditures. Although claims decreased by 14.8% (from 546 026 in 2013 to 465 089 in 2022), total payments increased by 60.5% (95% CI, 50.9%-90.6%; *P* < .001). Adjusting for medical care inflation, Medicare per-claim payments grew significantly by 26.2% (95% CI, 10.5%-49.4%; *P* = .007) over the study period. When adjusted for prescription drug inflation, the increase was even higher at 32.9% (95% CI, 24.8%-47.3%; *P* < .001). These inflation-adjusted trends show that the rising costs of DMTs may not be fully explained by inflation alone, suggesting the influence of other factors driving the growing economic burden (Table 1; Figure 2).

### Route of DMT Administration Category Claims and Payment per Claim

Among the 63 DMTs analyzed, there were 17 oral, 27 injectable, and 19 infusible DMTs. From 2013 to 2022, oral DMT claims declined 55.4% (95% CI, −84.9% to −57.7%; *P* < .001), while payment per claim increased by 50.4% (95% CI, 36.7%-69.4%; *P* < .001), 18.2% (95% CI, 0.1%-32.2%; *P* = .049) after medical care inflation, and 24.5% (95% CI, 13.1%-30.6%; *P* < .001) after prescription drug inflation. Injectable DMTs claims changed by 58.0% (95% CI, −20.4% to 102.2%; *P* = .16), with payment per claim changing by 128.8% (95% CI, 23.5%-259.6%; *P* = .02), 79.9% (95% CI, −33.7% to 186.7%; *P* = .15) after medical care inflation, and 89.5% (95% CI, −11.1% to 182.5%; *P* = .08) after prescription drug inflation. Infusible DMTs claims increased 264.3% (95% CI, 280.0%-327.2%; *P* < .001), with payment per claim increasing 61.4% (95% CI, 60.7%-94.7%; *P* < .001), 26.9% after medical care inflation (95% CI, 24.8%-50.8%; *P* < .001), and 33.6% after prescription drug inflation (95% CI, 23.5%-64.2%; *P* = .001) (Table 2).

**Table 2.** All Claims and Payments Per Claim for Route of DMT Administration Category (63 Unique DMTs), 2013 to 2022

Administration category	No. of claims		Percentage change (95% CI)	*P* value	Mean (SD) Medicare payment per claim, $		Percentage change (95% CI)	*P* value	Adjusted mean (SD) Medicare payment per claim, $							
									Medical care inflation				Prescription drug inflation			
									Year		Percentage change (95% CI)	*P* value	Year		Percentage change (95% CI)	*P* value
	2013	2022			2013	2022			2013	2022			2013	2022		
Oral DMTs (n = 17)	314 626	140 492	−55.4 (−84.9 to −51.7)	<.001	4789.44 (239.90)	7202.50 (2485.70)	50.4 (36.7 to 69.4)	<.001	4789.44 (239.90)	5663.04 (1954.41)	18.2 (0.1 to 32.2)	.049	4789.44 (239.90)	5964.30 (2058.70)	24.5 (13.1 to 30.6)	<.001
Injectable DMTs (n = 27)	200 472	316 828	58.0 (−20.4 to 102.2)	.16	1496.36 (2309.47)	3423.85 (5029.73)	128.8 (23.5 to 259.6)	.02	1496.36 (2309.47)	2692.04 (3954.68)	79.9 (−33.7 to 186.7)	.15	1496.36 (2309.47)	2835.23 (4165.06)	89.5 (−11.1 to 182.5)	.08
Infusible DMTs (n = 19)	31 009	112 978	264.3 (280.0 to 327.2)	<.001	7779.11 (2489.96)	12 553.55 (6454.67)	61.4 (60.7 to 94.7)	<.001	7779.11 (2489.96)	9870.36 (5075.05)	26.9 (24.8 to 50.8)	<.001	7779.11 (2489.96)	10 395.44 (5345.03)	33.6 (23.5 to 64.2)	.001

Abbreviation: DMT, disease-modifying therapy.

### DMT Subclass Payments Per Claim

Of 57 unique DMTs analyzed (excluding corticosteroids), therapies were classified into 14 subclasses based on mechanism of action. In 2013, azathioprine had the lowest mean (SD) payment per claim ($36.36 [$29.08]), with immunoglobulins having the highest ($8606.86 [$1121.33]); by 2022, methotrexate was the least costly ($37.35 [$36.71]), with inebilizumab and eculizumab being the most costly ($38 993.14 [$8016.72]). Among FDA-approved therapies for multiple sclerosis, glatiramer acetate claims declined 55.4% (95% CI, −95.1% to −40.4%; *P* < .001), with a 7.8% (95% CI, −22.2% to 27.7%; *P* = .81) unadjusted change in payment per claim but a 15.3% decrease after adjusting for medical care inflation (95% CI, −45.7% to −2.0%; *P* = .04). Interferon β-1 claims decreased 69.4% (95% CI, −91.1% to −71.2%; *P* < .001), while payment per claim increased 60.6% (95% CI, 60.1%-72.6%; *P* < .001). Azathioprine claims decreased numerically (−8.4%; 95% CI, −47.3% to 12.7%; *P* = .22), although the change was not statistically significant, yet payment per claim increased 179.3% (95% CI, 122.0%-237.1%; *P* < .001), remaining among the lowest cost DMTs in absolute terms. High-cost therapies expanded markedly, with inebilizumab and eculizumab claims increasing by 1638.3% from 2018 to 2022 (95% CI, 1196.4%-2988.6%; *P* = .001) despite a 5.0% payment reduction (95% CI, −7.7% to −2.2%; *P* = .001), and other DMTs for multiple sclerosis (including alemtuzumab and natalizumab) rose 314.3% in claims (95% CI, 99.5%-605.0%; *P* = .01) with a 145.6% increase in payment per claim (95% CI, 144.5%-177.2%; *P* < .001). Although not significant, the Nrf2 activator class exhibited a 0.6% decrease in payment per claim (95% CI, −38.9% to 108.1%; *P* = .31) and an even more pronounced decrease after adjustment for medical care inflation (21.8%; 95% CI, −59.6% to 63.8%; *P* = .94). Anti–CD-20 therapy increased significantly by 42 800% (95% CI, 43 254%-58 135.4%; *P* = .03), as did immunoglobulin therapy (300.2%; 95% CI, 323.7%-389.5%; *P* < .001) (Table 3).

**Table 3.** Payments Per Claim for 14 Classes of DMTs, Excluding Corticosteroids (57 Unique DMTs), 2013 to 2022

Subclass	No. of claims		Percentage change (95% CI)	*P* value	Mean (SD) Medicare payment per claim, $		Percentage change (95% CI)	*P* value	Adjusted mean (SD) Medicare payment per claim, $							
									Medical care inflation				Prescription drug inflation			
									Year		Percentage change (95% CI)	*P* value	Year		Percentage change (95% CI)	*P* value
	2013	2022			2013	2022			2013	2022			2013	2022		
Glatiramer acetate (n = 3)	164 669	140 492	−55.4 (−95.1 to −40.4)	<.001	4980.68 (602.58)	5366.49 (1623.87)	7.8 (−22.2 to 27.7)	.81	4980.68 (602.58)	4219.45 (1276.79)	−15.3 (−45.7 to −2.0)	.04	4980.68 (602.58)	4219.45 (1344.71)	−10.8 (−34.9 to −4.3)	.02
Interferon β-1 (n = 8)	149 703	45 788	−69.4 (−91.1 to −71.2)	<.001	4574.69 (152.89)	8872.64 (831.71)	94.0 (94.8 to 121.7)	<.001	4574.69 (152.89)	6976.21 (653.95)	52.5 (44.6 to 75.5)	<.001	4574.69 (152.89)	7347.32 (688.73)	60.6 (60.1 to 72.6)	<.001
Anti–CD-20 therapies (n = 3)^a^	35	15 015	42 800.0 (4325.4 to 58135.4)	.03	8236.74 (0)	10 165.98 (4783.70)	23.4 (−71.2 to 591.0)	.11	8236.74 (0)	8377.60 (3942.57)	1.7 (−87.6 to 521.2)	.14	8236.74 (0)	9013.10 (4241.62)	9.4 (−69.7 to −7.2)	.12
Immunoglobulin (n = 14)	27 057	108 294	300.2 (323.7 to 389.5)	<.001	8606.86 (1121.33)	11 477.92 (1783.82)	33.4 (30.3 to 51.1)	<.001	8606.86 (1121.33)	9024.63 (1402.54)	4.9 (−0.3 to 16.4)	.06	8606.86 (1121.33)	9504.72 (1477.16)	10.4 (−2.4 to 28.6)	.09
Azathioprine (n = 3)	24 327	22 278	−8.4 (−47.3 to 12.7)	.22	36.36 (29.08)	101.52 (50.12)	179.3 (122.0 to 237.1)	<.001	36.36 (29.08)	79.82 (42.18)	119.6 (57.7 to 166.8)	<.001	36.36 (29.08)	84.07 (45.13)	131.2 (79.7 to 168.4)	.001
Nrf2 activators (n = 4)	17 297	64 754	274.4 (−316.5 to 464.0)	.68	5259.61 (785.52)	5230.38 (3468.78)	−0.6 (−38.9 to 108.1)	.31	5259.61 (785.52)	4112.40 (2727.36)	−21.8 (−59.6 to 63.8)	.94	5259.61 (785.52)	4331.20 (2872.45)	−17.7 (−48.4 to 61.0)	.80
S1P receptors (n = 4)	30 701	25 126	−18.2 (−68.1 to 0.2)	.051	5318.40 (785.52)	10 212.74 (521.09)	92.0 (90.8 to 117.6)	<.001	5318.40 (785.52)	8029.90 (409.23)	51.0 (41.8 to 72.4)	<.001	5318.40 (785.52)	8457.04 (431.00)	59.0 (58.6 to 70.0)	<.001
Methotrexate (n = 5)	6262	3723	−40.6 (−67.8 to −33.9)	<.001	50.94 (2.63)	37.35 (36.71)	−26.9 (−80.7 to −17.3)	.007	50.94 (2.63)	29.30 (28.86)	−42.5 (−95.3 to −37.7)	.001	50.94 (2.63)	30.90 (30.40)	−39.4 (−91.7 to −35.0)	.001
Mycophenolate (n = 3)	21 939	37 358	70.3 (60.2 to 94.2)	<.001	173.61 (228.44)	162.93 (99.44)	−6.2 (−15.8 to 12.4)	.79	173.61 (228.44)	128.10 (78.18)	−26.2 (−37.3 to −10.9)	.003	173.61 (228.44)	134.92 (82.34)	−22.3 (−39.1 to −2.6)	.03
TNF-α (n = 1)^b^	11	52	381.8 (312.7 to 1020.9)	.002	4625.46 (0)	5833.18 (0)	26.1 (19.2 to 168.5)	.02	4625.46 (0)	4683.00 (0)	1.3 (−12.3 to 138.1)	.09	4625.46 (0)	4898.20 (0)	5.9 (−6.7 to 141.7)	.07
Inebilizumab and eculizumab (n = 2)^c^	287	4989	1638.3 (1196.4 to 2988.6)	.001	41 043.67 (0)	38 993.14 (8016.72)	−5.0 (−7.7 to −2.2)	.001	41 043.67 (0)	35 058.61 (7207.80)	−14.6 (−22.0 to −7.7)	.002	41 043.67 (0)	38 679.70 (7952.27)	−5.8 (−21.9 to −2.6)	.001
Other DMTs for multiple sclerosis (n = 3)	11 799	48 887	314.3 (99.5 to 605.0)	.01	4253.75 (54.82)	10 448.27 (488.70)	145.6 (144.5 to 177.2)	<.001	4253.75 (54.82)	8215.16 (4315.54)	93.1 (83.5 to 118.6)	<.001	4253.75 (54.82)	8652.08 (4315.54)	103.4 (96.1 to 124.0)	<.001
Bortezumab (n = 1)^d^	21	11	−47.6 (−74.3 to 116.2)	.63	3292.38 (0)	5549.89 (0)	68.6 (−65.9 to 266.7)	.20	3292.38 (0)	4711.00 (0)	43.1 (−67.4 to 236.5)	.24	3292.38 (0)	5067.50 (0)	53.9 (−65.4 to 246.3)	.22
IL-6 inhibitor (n = 2)^e^	282	651	130.9 (15.6 to 321.5)	.005	15 934.30 (2401.52)	16 138.31 (3800.54)	1.2 (−15.7 to 179.5)	.95	15 934.30 (2401.52)	12 688.90 (3708.80)	−2.0 (−12.3 to 143.1)	.93	15 934.30 (2401.52)	15 937.81 (3753.32)	0.2 (−13.0 to 149.3)	.99

Abbreviations: DMT, disease-modifying therapy; IL-6, interleukin 6; S1P, sphingosine-1-phosphate; TNF-α, tumor necrosis factor α.

^a^ Year of first recorded Medicare Part D claim, 2015.

^b^ Year of first recorded Medicare Part D claim, 2014.

^c^ Year of first recorded Medicare Part D claim, 2018.

^d^ Year of first recorded Medicare Part D claim, 2016.

^e^ Year of first recorded Medicare Part D claim, 2021.

Teriflunomide showed the largest increase in payments per claim, which rose by 138.4% (95% CI, 129.7%-171.6%; *P* < .001) in 2022. The inflation-adjusted increases remained significant, with payments per claim growing by 87.5% (95% CI, 68.0%-115.4%; *P* < .001) after adjusting for medical care inflation and by 97.4% (95% CI, 84.6%-116.1%; *P* < .001) after adjusting for prescription drug inflation (eTable 2 in Supplement 1).

## Discussion

This economic evaluation found that from 2013 to 2022, DMTs for immune-mediated neurologic diseases accounted for 2.4% of Medicare Part D claims for neurologic conditions but for 40.8% of total Medicare spending on neurologic drugs, highlighting the substantial financial contribution of DMTs within federally funded insurance claims. During this period, the total annual number of DMT claims increased modestly by 3.8%, while Medicare’s annual expenditure on these therapies rose substantially by 70.3%. The mean payment per claim increased significantly by 64.2%, even after adjustments for medical care and prescription drug inflation. This rise remained significant, showing at least a 29% increase above 2013 levels across all DMT analyses (Table 1). Similarly, for DMTs that remained continuously available, the inflation-adjusted increase remained significant, with a minimum rise of 26%. These findings suggest that increases in payment per claim may not be solely attributable to inflation, consistent with prior reports of rising prescription costs for neurologic conditions.^1,20^ Other cost drivers not captured by inflationary adjustments may include drug-specific shortages of raw materials or components, as well as isolated labor or supply chain expenses. However, if these factors were the primary contributors, they would be expected to be associated with sporadic, rather than consistent, sustained increases across all DMT classes.

Over the past decade, claims for oral DMTs decreased by 55.4%, while claims for injectable DMTs increased by 58.0% and claims for infusible DMTs by 264.3%. The decline in oral DMT claims may be attributed to the classification of most oral DMTs as immunomodulatory rather than immunosuppressive, particularly for multiple sclerosis. This trend represents a shift in the treatment paradigm for multiple sclerosis, moving from an escalation approach to early aggressive DMT strategies.^21^ The growth of infusible DMTs may be partly attributed to increased recognition of autoimmune neurologic disorders (eg, autoimmune encephalitis, stiff person spectrum disorders) and the expanded use of infusible anti–CD-20 therapies and immunoglobulins.^14,15,22,23,24,25^ Over the analysis period, claims specifically for anti–CD-20 therapies significantly increased 428-fold, while claims for immunoglobulins tripled (Table 3). The rise in anti–CD-20 therapy claims may be driven, in part, by the FDA’s approval of both infusible and injectable formulations for multiple sclerosis, which expanded therapeutic options and accessibility.^26,27,28,29,30,31^

Our analysis of payment per claim across DMT classes revealed some notable trends. Methotrexate had the largest decline at 26.9%, while azathioprine showed the most substantial increase, 179.3% over the past decade, not adjusting for inflation. A separate analysis of azathioprine, available in the US since 1968, showed a 900% increase in payment per claim through January 2023.^32^ This upward trend in azathioprine cost underscores an overall rise in payment rates over time. Among FDA-approved DMTs for multiple sclerosis, the Nrf2 activator class exhibited a 0.6% decrease in payment per claim. However, when adjusted for inflation, this decline was more pronounced at 21.8%. This reduction may be attributed to the FDA approval of a generic dimethyl fumarate in 2020.^33^ Our analysis revealed a marked decrease in payment per claim for this class, from $9541.73 in 2020 to $6945.62 in 2021 and $5230.38 in 2022. These findings suggest a notable cost-savings associated with the availability of generic products, which not only contribute to cost reduction but also highlight how the introduction of generics may influence market dynamics by reducing payments. In contrast, the other DMTs for multiple sclerosis, including alemtuzumab, cladribine, teriflunomide, and natalizumab, had a 145% increase in payment per claim over the study period. Among these therapies, only cladribine received recent FDA approval in 2019^34^ and accounted for less than 1% of claims and 5.6% of the payment within this DMT class between 2019 and 2022, suggesting a limited impact on overall payment trends. The other 3 DMTs (alemtuzumab, teriflunomide, and natalizumab) have been commercially available in the US since 2012 to 2014.^35,36,37^ These findings suggest that the overall increase in payment per claim within the other DMTs for multiple sclerosis class was not primarily driven by introducing innovative DMTs but may represent ongoing economic factors, such as pricing adjustments or demand for established therapies.

A focused analysis of the top 5 DMTs with the largest claims increase from 2013 to 2022 also revealed notable trends in use and cost. Among these, intravenous immunoglobulin exhibited the most pronounced growth in claims, increasing nearly 11-fold over the study period. Notably, immunoglobulins were 2 of the top 5 DMTs, with the largest increases in claims. This trend may reflect the growing recognition and diagnosis of autoimmune neurologic disorders, as well as an increased reliance on immunoglobulins in disease management. These findings suggest a potential shift in clinical practice patterns and therapeutic strategies in response to an evolving understanding of immune-mediated neurologic diseases. This substantial rise highlights an increasing reliance on intravenous immunoglobulin for managing immune-mediated neurologic disorders, potentially reflecting expanded indications, heightened clinician familiarity, or broader patient accessibility. Teriflunomide showed the largest increase in payment per claim, which rose by 138.4% (from $4226.64 in 2013 to $10 077.08 in 2022). The medical care and prescription drug inflation–adjusted increases remained significant at 87.5% and 97.4%, respectively. These findings suggest that the increase in cost may not have been solely driven by inflation but may reflect changes in pricing strategies, market dynamics, or associated health care costs.

Disease-modifying therapy costs for immune-mediated neurologic diseases have risen substantially, outpacing both medical care and prescription drug inflation. This trend has persisted even among continuously available agents, reflecting sustained increases in payment per claim that place considerable pressure on the Medicare budget. As Medicare represented 29% of national health care expenditures in 2022,^38^ the ongoing escalation in DMT costs poses an important challenge to the sustainability of federally funded health care for neurologic conditions. Notably, DMTs for immune-mediated neurologic diseases accounted for more than 40% of neurologic disease Medicare Part D expenditures in the past decade, underscoring their disproportionate financial impact compared with other treatment categories. The Inflation Reduction Act, which includes provisions for the Medicare Drug Price Negotiation Program, represents a potential pathway to mitigate these rising costs.^39^ By enabling Medicare to negotiate the prices of high-cost drugs, the Medicare Drug Price Negotiation Program presents a promising opportunity to reduce the financial burden of DMTs on Medicare Part D and improve access to these essential therapies for patients with immune-mediated neurologic diseases. However, one of the most opaque and influential contributors to drug spending is the role of pharmacy benefit managers, whose complex rebate structures and profit mechanisms remain largely hidden from public oversight. In 2021, the gross-to-net bubble, ie, the difference between drug list prices and net prices after rebates and discounts, reached $204 billion, underscoring the scale of nontransparent pricing within the US health care system.^40^ A 2024 Federal Trade Commission staff report found that the 3 largest pharmacy benefit managers, CVS Caremark, Express Scripts, and OptumRx, generated more than $7.3 billion in revenue from marking up specialty drugs at affiliated pharmacies between 2017 and 2022 and an additional $1.4 billion from spread pricing.^41^ This lack of transparency extends to drug manufacturers, whose limited disclosure of net pricing and revenue generation impedes a full understanding of the true economic impact of these therapies. While rewarding innovation and investment in research and development is essential, such rewards should not persist indefinitely, particularly when they stifle competition or delay access to generics and biosimilars. Sustainable reform in Medicare Part D would require improved transparency across the supply chain, including pharmacy benefit managers and manufacturers, to ensure equitable access to high-cost therapies without compromising innovation.

### Strengths and Limitations

A strength of this analysis is the comprehensive assessment of decade-long payment trends in DMTs for immune-mediated neurologic diseases under Medicare Part D. However, several limitations should be considered. First, the Medicare Part D database represents a single insurance payer, and the payment trends identified herein may not fully reflect those of other payers. Despite this limitation, similar findings were noted in a recent analysis using the MarketScan database, which includes patients with predominantly private insurance.^20^ That analysis revealed a 217% increase in out-of-pocket costs for DMTs for multiple sclerosis from 2012 to 2021, suggesting a broader trend of rising costs across different insurance types. Second, our study lacked granular data on patient disease characteristics and progression, which may influence neurologists’ prescribing patterns and contribute to DMT costs. Because our analysis accounts for gross spending but not for manufacturer rebates, actual net costs may be modestly lower. However, prior research has shown that biologic and immunologic therapies, including most of the DMTs analyzed here, typically receive rebates of less than 10%,^42^ suggesting that the observed trends and increases in Medicare Part D expenditures would remain substantial. Finally, Medicare Part D is primarily used by individuals aged 65 years or older, whereas many immune-mediated neurologic diseases, including multiple sclerosis, are more common in younger populations.^14,43,44^ However, a substantial proportion (approximately 25%-30%) of Medicare beneficiaries are younger individuals who qualify due to disability, meaning that a substantial portion of the overall patient population is represented within this dataset. These limitations underscore the importance of considering broader insurance datasets and clinical characteristics in future studies to enhance understanding of DMT cost trends across broad populations.

## Conclusions

This 10-year economic evaluation of Medicare Part D claims for DMTs targeting immune-mediated neurologic diseases showed that DMT payments increased substantially, far outpacing inflation. While total claim volume rose modestly, payments grew significantly, particularly for infusible therapies. Costs remained elevated even after adjusting for medical care and prescription drug inflation. Payment trends varied by administration route and therapeutic class, with infusible DMTs showing the largest increases. These findings underscore the disproportionate economic burden of DMTs, accounting for more than 40% of Medicare Part D spending on neurologic conditions, and highlight the need for continued cost monitoring and potential policy interventions, such as the Medicare Drug Price Negotiation Program, beyond manufacturer rebates.

## Notes

Supplement 1. **eTable 1.** List of DMTs for Immune-Mediated Neurologic Diseases Included in the Analysis, Organized by Route of Administration **eTable 2.** Changes in Claims and Payment Per Claim for the Tope 5 DMTs With the Largest Increases in Claims (2013-2022)

Supplement 2.
Data Sharing Statement
