# Supplementary material for: Medicare Part D Use and Costs for Immune-Mediated Neurologic Therapies
Source: JAMA Netw Open. 2025 Oct 20;8(10):e2538277. doi: 10.1001/jamanetworkopen.2025.38277 (PMC12538365; doi:10.1001/jamanetworkopen.2025.38277)
Supplement: Supplement 1. — eTable 1. List of DMTs for Immune-Mediated Neurologic Diseases Included in the Analysis, Organized by Route of Administration eTable 2. Changes in Claims and Payment Per Claim for the Tope 5 DMTs With the Largest Increases in Claims (2013-2022) [file jamanetwopen-e2538277-s001.pdf]

Supplemental Online Content

Wong KH, Zeng E, Smith TL, et al. Medicare Part D use and costs for immune-mediated neurologic therapies. *JAMA Netw Open*. 2025;8(10):e2538277. doi:10.1001/jamanetworkopen.2025.38277

eTable 1. List of DMTs for Immune-Mediated Neurologic Diseases Included in the Analysis, Organized by Route of Administration
eTable 2. Changes in Claims and Payment Per Claim for the Top 5 DMTs With the Largest Increases in Claims (2013-2022)

This supplemental material has been provided by the authors to give readers additional information about their work.

© 2025 Wong KH et al. *JAMA Network Open*.

eTable 1. List of DMTs for Immune-Mediated Neurologic Diseases Included in the Analysis, Organized by Route of Administration		
**Injectable- Brand (Generic)**	**Infusible- Brand (Generic)**	**Oral (Brand and Generic)**
Avonex (Interferon beta-1a)	Actemra Actpen (Tocilizumab)	Aubagio (Teriflunomide)
Avonex Pen (Interferon beta-1a)	Bivigam (Immune Globulin Intravenous)	Azasan (Azathioprine)
Betaseron (Interferon beta-1b)	Cyclophosphamide (Cyclophosphamide)	Azathioprine (Azathioprine)
Copaxone (Glatiramer Acetate)	Flebogamma Dif (Immune Globulin Intravenous)	Bafiertam (Monomethyl Fumarate)
Cuvitru (Immune Globulin Subcutaneous)	Gammagard Liquid (Immune Globulin Intravenous)	Cellcept (Mycophenolate Mofetil)
Cutaquig (Immune Globulin Subcutaneous)	Gammagard S-D (Immune Globulin Intravenous)	Dimethyl Fumarate (Dimethyl Fumarate)
Enspryng (Satralizumab)	Gammaked (Immune Globulin Intravenous)	Gilenya (Fingolimod)
Extavia (Interferon beta-1b)	Gammaplex (Immune Globulin Intravenous)	Imuran (Azathioprine)
Glatiramer Acetate (Glatiramer Acetate)	Gamunex-C (Immune Globulin Intravenous)	Mavenclad (Cladribine)
Glatopa (Glatiramer Acetate)	Ocrevus (Ocrelizumab)	Mayzent (Siponimod)
Hizentra (Immune Globulin Subcutaneous)	Octagam (Immune Globulin Intravenous)	Methotrexate (Methotrexate)
Hyqvia (Immune Globulin Subcutaneous + Hyaluronidase)	Privigen (Immune Globulin Intravenous)	Methotrexate Sodium (Methotrexate Sodium)
Kesimpta Pen (Ofatumumab)	Remicade (Infliximab)	Mycophenolate Mofetil (Mycophenolate Mofetil)
Plegridy (Peginterferon beta-1a)	Rituxan (Rituximab)	Mycophenolic Acid (Mycophenolic Acid)
Plegridy Pen (Peginterferon beta-1a)	Soliris (Eculizumab)	Otrexup (Methotrexate Sodium)
Rebif (Interferon beta-1a)	Solu-Medrol (Methylprednisolone)	Ponvory (Ponesimod)
Rebif Rebidose (Interferon beta-1a)	Tysabri (Natalizumab)	Prednisolone (Prednisolone)
	Xembify (Immune Globulin Subcutaneous)	Prednisolone Acetate (Prednisolone Acetate)
		Prednisolone Sodium Phosphate (Prednisolone Sodium Phosphate)
		Prednisone (Prednisone)
		Prednisone Intensol (Prednisone)
		Rasuvo (Methotrexate)
		Tecfidera (Dimethyl Fumarate)
		Trexall (Methotrexate)
		Vumerity (Diroximel Fumarate)
		Xeljanz (Tofacitinib)
		Zeposia (Ozanimod)

© 2025 Wong KH et al. *JAMA Network Open*.

eTable 2. Changes in Claims and Payment Per Claim for the Top 5 DMTs With the Largest Increases in Claims (2013-2022)																
	Number of Claims				Average (SD) Medicare Payment per Claim				Medical Care Inflation Adjusted Average (SD) Medicare Payment per Claim				Prescription Inflation Adjusted Average (SD) Medicare Payment per Claim			
	Year 2013	Year 2022	% change [95% CI]	p-Value	Year 2013	Year 2022	% change [95% CI]	p-Value	Year 2013	Year 2022	% change [95% CI]	p-Value	Year 2013	Year 2022	% change [95% CI]	p-Value
**Aubagio (Teriflunomide)**	9,480	44,771	372.3% [120.6, 726.4]	p=0.01	$4,226.64 (586.77)	$10,077.08 (1868.08)	138.4% [129.7, 171.6]	p<0.001	$4,226.64 (586.77)	$7,923.21 (1468.80)	87.5% [68.0, 115.4]	p<0.001	$4,226.61 (586.77)	$8,344.7 (1546.93)	97.4% [84.6, 116.1]	p<0.001
**Prednisone (Steroids)**	90,000	118,358	31.5% [19.5, 46.6]	p=0.001	$6.61 (3.31)	$10.23 (6.18)	54.8% [-18.2, 68.2]	p=0.21	$6.61 (3.31)	$8.05 (4.86)	21.7% [-54.4, 30.3]	p=0.52	$6.61 (3.31)	$8.47 (5.12)	28.2% [-47.0, 34.8]	p=0.73
**Gamunex-C (Immunoglobulin)**	8,676	31,959	268.4% [274.9, 381.5]	p<0.001	$7,320.81 (5145.19)	$9,359.53 (6024.57)	27.8% [18.1, 47.4]	p=0.001	$7,320.81 (5145.19)	$7,359.00 (4736.88)	0.5% [-10.7, 14.1]	p=0.76	$7,320.81 (5145.19)	$7,750.51 (4988.88)	5.9% [-12.7, 25.9]	p=0.45
**Mycophenolate Mofetil**	21,311	37,038	73.8% [63.9, 99.2]	p<0.001	$134.40 (77.14)	$156.67 (158.98)	16.6% [11.1, 35.9]	p=0.002	$134.40 (77.14)	$123.2 (125.00)	-8.3% [-15.5, 5.6]	p=0.33	$134.40 (77.14)	$129.73 (131.65)	-3.5% [-15.8, 14.9]	p=0.95
**Privigen (Immunoglobulin)**	1,336	16,000	-1,097.6% [1198.4, 1705.6]	p<0.001	$9,119.85 (7043.68)	$11,757.00 (7873.96)	28.9% [22.6, 49.4]	p<0.001	$9,119.85 (7043.68)	$9,244.06 (6190.98)	1.4% [-7.7, 17.3]	p=0.41	$9,119.85 (7043.68)	$9,735.82 (6520.33)	6.8% [-0.4, 19.8]	p=0.06

© 2025 Wong KH et al. *JAMA Network Open*.
