# Supplementary material for: Medicare Part D Use and Costs for Immune-Mediated Neurologic Therapies
Source: JAMA Netw Open. 2025 Oct 20;8(10):e2538277. doi: 10.1001/jamanetworkopen.2025.38277 (PMC12538365; doi:10.1001/jamanetworkopen.2025.38277)
Supplement: Supplement 2. — Data Sharing Statement [file jamanetwopen-e2538277-s002.pdf]

Data Sharing Statement

Data
Data available: Yes
Data types: Data (not involving human participants)
How to access data: Data will be made available upon request
When available: With publication

Supporting Documents
Document types: Statistical/analytic code
How to access documents: It will be made available upon request
When available: With publication

Additional Information
Who can access the data: Will be made available upon request
Types of analyses: Statistical coding
Mechanisms of data availability: All investigators upon request
